# ConSQL: A Database Language for Contex-aware Conversational Cooperative Query Answering

**DOI:** 10.21203/rs.3.rs-3129882/v1

**Published:** 2023-07-13

**Authors:** Hasan Jamil

**Affiliations:** Department of Computer Science, University of Idaho, 875 Perimeter Drive, Moscow, 83844, ID, USA

**Keywords:** Contextual querying, query relaxation, non-monotonic constraint inheritance, preferred tuples, top-***k*** querying

## Abstract

In conversational query answering systems, context plays a significant role in accurately and meaningfully carrying it forward. In many chatbots, such as in Expedia, the discussion quickly degenerates into circling back to restarting the conversation or to inviting a live agent to intervene because the bot could not grasp the context. Contexts shorten interactions by way of implied query constraints to narrow search and to not repeat them in subsequent queries. In this paper, we introduce a novel way of viewing contexts as a distance function via the concept of query relaxation. We demonstrate that a typed domain distance function is sufficient to model context in a conversation. Our approach is based on the idea of non-monotonic constraint inheritance in a context hierarchy.

## 1 Introduction

The concept of context is often application specific and a general definition of it is not available. Early research in database (DB) and artificial intelligence (AI) context has been modeled sometimes indirectly without an explicit treatment. For example, cooperative query answering [1, 2], indefinite and maybe queries [3], incomplete databases [4], null values [5, 6], disjunctive databases [7, 8], contextual query answering [9, 10] and knowledge representation and reasoning [11–13]. Except for ConteLog [12], most of these systems treat context indirectly and at times, as a by product.

For example, consider the query against the table *Restaurants* below.

*Q*_1_: Is there a five star rated Vietnamese restaurant in Moscow, Idaho?

**Table:** 

TiD	Name	Location	Cuisine	Rating	Price	Dining

*t*_1_	Pho Heaven	Moscow	Vietnamese	*	Inexpensive	Carryout
*t*_2_	Hot Pot	Moscow	Korean	****	Very Expensive	Premise
*t*_3_	Ao Dai	Pullman	Vietnamese	*****	Moderate	Both
*t*_4_	Ravi Kabob	Arlington	Pakistani	*****	Inexpensive	Both
*t*_5_	Little Tibet	Spokane	Tibetan	*****	Expensive	Both

**Fig. 1:**
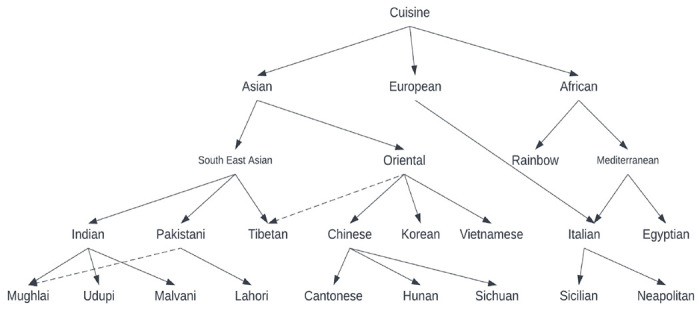
CoBase type abstraction hierarchy.

In a commonsense world, it is possible to imagine multiple equally acceptable responses depending on how one interprets the intent of the query *Q*_1_. In CoBase [1], the constants “Moscow”, “Vietnamese” and “*****” serve as the contexts in three different axes. Using a type abstraction hierarchy (TAH), the attribute domain values, or objects, are organized in a proximity hierarchy from fine to coarse as shown in Fig 1. In this hierarchy, “Vietnamese”, “Korean” and “Chinese” are shown to be similar (“Oriental”) types of cuisines. Therefore, they are also substitutable. “Tibetan” cuisine, on the other hand, is almost Oriental being close to Chinese cultural influence. “Mughlai” cusisine, however, is part of both “Indian” and “Paksitani” cuisine. Therefore, it can be included in a query response for Pakistani food.

The idea is that we relax the constraint (i.e., *Cuisine=”Vietnamese”*) by moving up the hierarchy one level, and determine that Korean food is also a possible match. We have a choice of computing the *similarity* of the cuisines using a suitable distance function – we can consider Vietnamese restaurants in rows *t*_1_ and *t*_3_ to be perfect matches, and Korean restaurants in row *t*_2_ to be a 99% match. This is because the height at which they match is at level 1 of a 3 tier high tree. In the absence of the dotted line showing membership of Tibetan cuisine in Oriental category, the similarity of Tibetan cuisine to Vietnamese will be 66% being within the group at height 2, and European” cuisine will be 33%.

Using similar TAH and similarity computing schemes, we can compute the similarities of the domain values in columns *Rating* and *Location*, and finally compute a composite similarity score for each tuple based on these three attributes. We can even accommodate a weighted similarity score using user assigned relative importance of each of these query conditions. While the idea is simple, and processing such queries is not too difficult, the construction and maintenance of the TAHs, however, are extremely complex. A slight change in membership often requires expensive reorganization. We refer the readers to [14] for a more detailed discussion on cooperative query answering in general and query relaxation in particular. Conceptually similar but tangential treatment of contexts are discussed in null value based [5, 6], disjunctive value based [7, 8] and maybe [3] type systems which try to guess the unknown column values against the query constants.

## 2 Related Research

While the emergence of ChatGPT has changed the way we query information repositories, flexible query answering using such large language models (LLM) is an emerging research area. ChatGPT-like systems are impressive in how they are able to keep the context of a conversation in focus, and often allow deviation from a chain of thought [15]; however, cooperative or flexible query answering is probably not their forte yet. In particular, they still are systems that ingest information from text documents (possibly from a corpora of archived text documents), and they usually do not ingest information from the hidden web databases [16] to function. At least, we are unaware of any research that attempted doing so yet.

To illustrate, let us pose same query *Q*_1_ to ChatGPT. The complete interaction can be found in this link ^1^. We first asked,

$Q_{1}^{\prime }$: Do you think you could suggest a 5 star Indian restaurant in Moscow, Idaho?

to which it basically responded by saying

I’m sorry, but I couldn’t find any information regarding a 5-star Indian restaurant in Moscow, Idaho. Moscow is a small city located in the state of Idaho, and it may not have a wide range of Indian dining options. However, there might be Indian restaurants or other dining establishments that serve Indian cuisine in the area. I recommend checking online review platforms, such as Yelp or Google Maps, for the most up-to-date information on Indian restaurants in Moscow, Idaho, and reading reviews to determine which one suits your preferences.

We then followed up by asking,

$Q_{1}^{\prime \prime }$: How about a five star Vietnamese restaurant?

to which it again responded similarly. The interesting part of this conversation is that ChatGPT did not miss the context even though the query about Vietnamese restaurant was posed in a different way. Like a human would, it remembered or could identify, the context accurately. Finally, even when we supplied the table in Sec 1 and asked question $Q_{1}^{\prime \prime }$ a final time, ChatGPT responded by saying

Based on the provided table, “Pho Heaven” in Moscow, ID, is listed as a Vietnamese restaurant with a star rating of 1. However, there are no five-star Vietnamese restaurants listed in Moscow, ID. Therefore, based on the given information, there isn’t a specific five-star Vietnamese restaurant recommendation in Moscow, ID.

and failed to suggest an alternative.

However, as discussed in Sec 1, we prefer that a cooperative system such as CoBase return Tibetan restaurant, “Little Tibet” in Spokane, “Ao Dai” Vietnamese restaurant in Pullman, or “Hot Pot” Korean restaurant in Moscow as possible alternative responses. This is mainly because LLM based query answering systems are primarily text driven, not database driven, especially not deep web database driven. It is also not yet truly possible for these systems to approximate responses objectively without any help from additional structures. For example, when prompted for a best approximation of query $Q_{1}^{\prime \prime }$ as an alternative, it suggested the following:

In the absence of a five-star Vietnamese restaurant in Moscow, ID, “Ao Dai” in Pullman, WA could be a good alternative option. It is listed as a Vietnamese restaurant with a star rating of 5. Although it is located in Pullman, which is approximately 8 miles away from Moscow, it might be worth considering if you are willing to travel a short distance for a highly rated Vietnamese dining experience.

which is one of the cooperative responses. Still ChatGPT did not suggest “Hot Pot” or “Little Tibet” even though they are other possible approximations. As a final thought, we asked, what if we remove rows 1 and 3, ChatGPT could not suggest any. This goes to highlight the fact that it only considers obvious, highly visible facts. Interestingly, it was able to determine location similarity without a TAH, which is significant, but failed to understand that Tibetan or Korean cuisines could be substitutes for Vietnamese cuisines.

It must be noted here that some estimates suggest that the hidden web or deep web is several hundred times larger [17] than the shallow web [18]. Therefore, it is imperative that we also have systems capable of generating cooperative responses from deep web databases because it is not possible to supply a database to LLMs (as we did above) in the hope of getting a partial, inaccurate cooperative response.

Contemporary chatbots, however, are performing well in many areas of interest such as e-commerce [19], product development [20], health services [21], etc. While a handful of research have attempted to capture conversational context [20, 22], most such applications limit context modeling to technologies such as decision tree, keyword recognition, and goal directed workflows, and fall back to live agents (e.g., Expedia), when rendering effective service seems impossible. Though a few research efforts have started to address the issue [23], we believe it is too early to fully know and understand how LLMs model contexts (which they do quite well), and learn what limitations they may have. The research presented in this paper is an attempt to combine both cooperative query answering and context modeling in conversational systems to improve query responses. Most importantly, we introduce the idea of contextual cooperative query answering over structured databases that LLMs are currently not addressing. However, efforts are underway for LLMs to access structured databases [24], and once successful, efforts to bring structured data under LLM context models [25, 26] will likely bear fruit.

## 3 Context as a First-Class Citizen for Query Answering

Context plays a significant role in DialogueCRN [11], context reasoning [13], contextual query refinement [27], and contextual knowledge based query answering [9] systems. Only in the recently proposed ConteLog [12] was context modeled by giving it a syntax and a direct semantic. However, the model and the semantics assigned can only capture limited capabilities with a significant degree of user involvement. For example, in ConteLog, contextual queries of the form

*Q*_2_: “Which side of the street will the Hall Building be if I came from Dorval Airport along the De Maisonneuve Boulevard in Montreal?”

can be asked and answered only if the location of the Hall Building is available relative to the streets, and the direction and position contexts are explicitly supplied in the query. However, in ways similar to CoBase’s TAH, users must supply context information for ConteLog to be able to tease out interesting responses, thus making the platform similarly restrictive and cumbersome. We argue that a more hands off, and intuitive context modeling is preferred.

### 3.1 The Concept of Context in a Conversation

Technically, we view a conversation between a human agent, say Abebi, and a robot or a computer agent, say the Expedia chatbot, as a series of queries by Abebi and efforts by Expedia to answer those at each step. Usually, a conversation is initiated by the human agent, and answered by the chatbot until the human agent is satisfied or the robot exhausts all possibilities. Responses are often approximate [28], and can only be computed if some form of adjustments to the query conditions are made, i.e., relaxation [14].

**Fig. 2:**
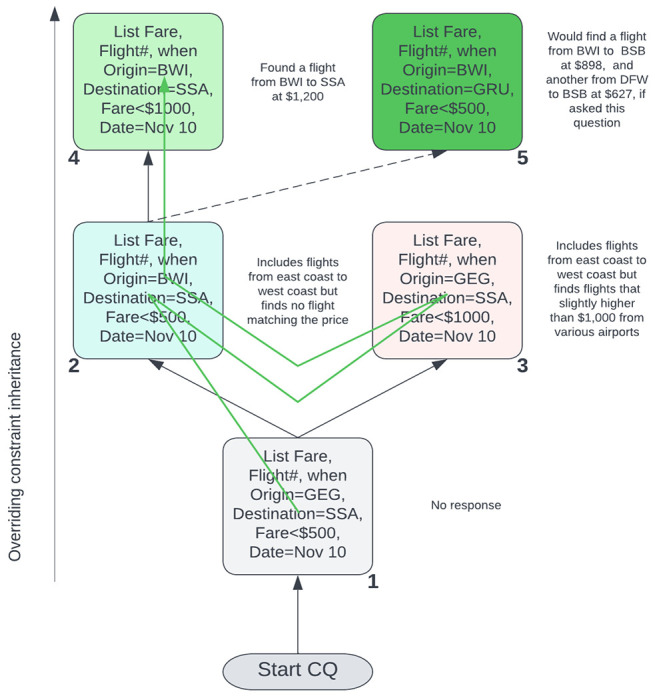
Inverted context tree as a stack.

In our view, the initial query conditions serve as the context. Each subsequent query is considered a relaxation of the initial context or conditions, and viewed as a stack of queries, the most recent being at the top. Given a series of queries the conditions that are not relaxed flow upward (stay unchanged or inherited), and the conditions that change, override the previous conditions. We call it an inverted inheritance stack-tree. We now discuss the idea using the example in Fig. 2. As shown, while queries can be asked in a single stack and contexts modified, it is also possible to *switch* contexts by opening branch stacks to explore multiple possibilities, and hop branches.

In the example in Fig. 2, the queries are asked in succession in the direction of the green arrow, and the description of responses that can be generated are shown on the side. In response to the initial query in the grey box number 1, no response could be found, and the *implied* context is set to the query conditions. The query response was empty largely because there were no flights from GEG (Spokane). However, as soon as the query in teal box 2 is asked by Abebi, in which the constraint *Origin* is relaxed from GEG to BWI (Baltimore), a significant number of flights became possible. However, the result is still empty. This is because by relaxing the *Origin* constraint, Abebi signaled that she is flexible on it and she is willing to accept a deviation up to a geographic distance from Spokane to Baltimore, which is about the entire continental USA in practical terms. However, the other constraints did not allow for a likely response.

The moment Abebi asked the query in the pink box 3 and relaxed the *Fare*, all the airports in USA with a fare less than $1,200 could now be searched. This relaxation allowed the discovery of the flight from BWI to SSA (Salvador) at $1,200 in the green box 4. It should be noted that there were cheaper flights on November 12, but were never considered because Abebi did not intend to relax the travel dates. On the other hand, Abebi never wanted to move the *Destination* from SSA to any other location to explore possibilities. Had she asked the question in the dark green box 5, she could have discovered the flights from BWI to BSB (Brasilia) at $898 and DFW (Dallas) to BSB at $627.

The guiding principle in our model is that every relaxation by the user establishes a wider set of possibilities with a target to select the closest ones stated in the initial query in the grey box 1 establishing a firm context that the user started with. The goal is to the find the tightest match. The mechanism used is *probing* in the form of query relaxation to indicate what and by how much deviation a user is willing to accept from the original stated intent. The constraints at any stage of the interrogation or conversation is carried forward in an overriding fashion (*Origin* in box 2 overrides the *Origin* in box 1). In box 3, the *Fare* overrides the *Fare* in box 1, but establishes a distinct context – a *context switch*.

### 3.2 Cumulative and Disjunctive Context Switch

The overall spirit captured in the conversation modeled in Fig. 2 in section 3.1 is that Abebi is looking for a flight from Spokane to Salvador on November 10 at a price less than $500, but she could fly from anywhere in the United States to anywhere near Salvador but not too far from Sao Paulo. She is also flexible on the fare up to about $1,200. What is not negotiable is the date of travel on November 10. The query context can also include a larger picture. For example, the profile of Abebi could serve as a more defining context, and sit at the bottom of the query stack to influence the exploration. Let’s assume that she is a Delta frequent flyer, has her home in Baltimore, and this a business trip for her paid for by her employer. It is also the case that Abebi will have to be in Baltimore after she returns from Salvador to spend her winter vacation. The question is, how do these sets of information change the search? Apparently, there are two distinct ways of applying the context based query refinement – *cumulative* and *disjunctive*. We now discuss these two semantics of contextual querying.

#### 3.2.1 Cumulative Context Switching

In cumulative context querying, users interactively explore a very large information space to discover a closest match to a target goal considered as the initial context. Every successive query asked, called the *probe* queries, is intended to steer the system to explore an altered information space with a set of relaxed constraints by treating the probes as multi-dimensional constraint relaxations. For example, the exploration in Fig. 2 can be seen as the following conversation in natural English.

1. Abebi: Could I have the fare for a flight from Spokane to Salvador on November 10 at a fare no higher than $500? – (*initial context*)
2. Agent: No, Madam, there are no such flights at this price on this day.
3. Abebi: Could you try it from Baltimore? – (*context switch from Spokane to Baltimore*)
4. Agent: There are numerous flights from many other airports not too far from Spokane, but none has a fare within your price range.
5. Abebi: How about at about $1,200 from Spokane? – (*context switch from Baltimore to Spokane and Price from $500 to $1,200*)
6. Agent: There is a Delta flight from Baltimore to Salvador at $1,200 on November 10.
  optionally, if asked
7. Abebi: Do you think I will get a cheaper fare close to $500 if I went to Sao Paulo instead? – (*context switch from Salvador to Sao Paulo and Price from $1,200 to $500 back*)
8. Agent: Yes, Ma’am. You can fly from Baltimore to Brasilia at $898 on American, or fly from Dallas-Forth Worth to Brasilia at $627 on Copa Airlines.

During the entire conversation above, the agent was squarely focused on finding the closest match to the constraints expressed in the initial context in step 1. Every time Abebi changed (relaxed) a constraint, the agent reconsidered the initial context and all the other relaxations to find the closest match so far using the maximum relaxation distance. This is the essence of cumulative context switching – the entire tree is focused on the initial context.

#### 3.2.2 Disjunctive Context Switching

In disjunctive context switching, on the other hand, the global view of the initial context is not preserved – only the top of the context stack in a single query stack remains the focus. For example, in the Fig. 2, box 2 and in the conversation in the previous section in step 3, the context *Origin* changed from Spokane to Baltimore, and it stayed in box 3 and in step 5 when the context *Price* changed keeping the *Origin* at Spokane. In disjunctive context switching, the relaxation in the other branch of the tree will not be active – only one branch at a time as a stack will be the active context. In other words, the agent’s response at step 6 will be

6a. Agent: There is no flight from Spokane to Salvador on November 10 at this price.

Note that the agent is not considering the relaxation of the *Origin* expressed in box 2 or step 3. Evidently, a disjunctive switch allows more selective and focused search suitable for weaving through an information space.

## 4 Contextual Query Language ConSQL

While a contextual conversational system can be implemented as a chatbot in natural English in text or voice, or as an interactive graphical interface, it will most likely interact with an SQL or NoSQL database in some fashion to carry out the search. In this section, we introduce an extension of SQL, called the ConSQL (pronounced consequel and stands for Contextual SQL) to model context in SQL.

### 4.1 Syntax of ConSQL

ConSQL supports conversations in one of the two contexts by a simple extension of basic SQL statements in a conversation tree similar to the one in Fig. 2. In fact, in a ConSQL database, conversations form a forest with multiple roots. The general syntax for a conversation has the following form.

begin [cumulative|disjunctive]|end|backtrack|fresh conversation;

A conversation begins with begin conversation instruction either in cumulative or in disjunctive mode. It can end with end conversation instruction. An entirely new conversation can be started from inside an active conversation with fresh conversation instruction. Conversations are unnamed sessions and are not persistent. Therefore, once a conversation is abandoned (using an end conversation instruction), it cannot be re-entered; it must start afresh. However, a new and additional initial context can be established by issuing fresh conversation instruction without ending a conversation, giving rise to conversation forests.

Once inside a conversation, a contextual query can begin. While a query generally has a traditional SQL syntax, it is annotated with a context modifier of the following form with a well-defined grammar.

 context *C*_1_ [parent *C*_2_] as

 select *A*_1_, *A*_2_ …, *A*_*n*_

 from *r*_1_, *r*_2_ … , *r_k_*

 where *θ*;

In the above form, *C*_1_ and *C*_2_ are conversation wide distinct identifiers^2^. The construction and issuance of these contextual queries must follow specific protocols and deserve a substantial discussion. However, in this article we want to be brief and only discuss essential components of ConSQL.

In a new or fresh conversation, the first contextual query must start with context *C* statement without the parent option since it must be the root, and the only context *C* statement until fresh conversation is issued. All subsequent statements must be of the form context *C* parent *C*′ to place the query under a node in the conversation or context tree. In both instances, a stack pointer points to the current context and all computations take place within its environment. To relocate the context pointer to alter the computational environment, backtrack conversation command can be issued to move the context toward the parent of the node. In the current edition of ConSQL, we do not see any need for a forward context pointer relocation particularly when there are multiple candidates, e.g., box 1 in Fig 2.

While several syntactic shortcuts are possible, we do not overburden the syntax of ConSQL to make querying easier since such shortcuts can be supported in the user interfaces in some fashion. For example, currently we require that the the query in step 3 be phrased in ConSQL as follows.

 *Q*_4_: context *C* parent *D* as

 select Flight*#*, Fare

 from *Flights*

 where *Origin=“BWI”* and *Destination=“SSA”* and *Fare<500* and *Date=11/10*;

where the initial context is set up as

 *Q*_3_: context *D* as

 select Flight*#*, Fare

 from *Flights*

 where *Origin=“GEG”* and *Destination=“SSA”* and *Fare<500* and *Date=11/10*;

Again, note that it is possible to imagine several fancy syntactic alternatives. Basically what we want in a modified context is to alter a condition in the parent context to relax it. Therefore, even if we allowed the syntax
 *Q*_5_: context *C* parent *D* as
 select Flight*#*, Fare
 from *Flights*
 where *Origin=“BWI”;*;
it would essentially mean the same query *Q*_4_ since it inherits all the unaltered constraints of *Q*_3_. In principle, we also allow adding new relations in the from clause in a subordinate context, i.e., monotonic inflation of information space so that all previously listed constraints can be enforced. For this reason also, we require the full syntax (not the *Q*_5_ form) not to make it difficult when mapping to SQL for execution and be counter intuitive.

### 4.2 Semantics of ConSQL

The semantics of ConSQL queries can be established based on the SQL queries with some fine tuning. In the current edition of ConSQL, we choose a tuple similarity based semantics for ConSQL in the following way.

**Definition 1** (Relaxation). *Given two conditions* $\theta_{1}$ and $\theta_{2}$ *over an attribute A in query Q*, $\theta_{2}$ *is called a relaxation or a* relaxed condition, *if* $e\left(Q_{\theta_{1}}\right)\subseteq e\left(Q_{\theta_{2}}\right)$, *where e is the evaluation function of query* $Q_{\theta_{i}}$ *with condition* $\theta_{i}$, *and i* ∈ {1, 2}.

#### 4.2.1 Distance Function

We assume that there is a type-polymorphic distance function $\delta^{t}$ that can compute the similarity of two identical typed data items. For example, numbers, GPS location based distance, star ratings, and even for complex types such as airports (similarity of LAX and AMS), and so on. For the types $\delta^{t}$ cannot compute the similarity, the distance will be undefined, i.e., ∞.

**Definition 2** (Close to Intent). *Let* $\theta_{2}$ *be a relaxation of* $\theta_{1}$ *over an attribute A of type t, and let c*_2_ *and c*_1_ *be the constants respectively. The closeness of a value v to the initial context or the intent is then given by*

$$\chi \left(v,c_{1},c_{2}\right)=\{\begin{array}{cc} 1 & if\;v\leq c_{1}\\ 1-\frac{\left(v-c_{1}\right)}{\left(c_{2}-c_{2}\right)} & if\;c_{1}<v\leq c_{2} \end{array}$$

Thus, given two values *v*_1_ and *v*_2_, *v*_1_ is closer to the intent than *v*_2_ if $\chi \left(v_{2},c_{1},c_{2}\right)\leq \chi \left(v_{1},c_{1},c_{2}\right)$. Using the definition of closer values, we now can construct the notion of preferred tuples as follows.

**Definition 3** (Preferred Tuples). *Let t is a tuple over the scheme* $A_{1},A_{2},\ldots ,A_{n}$. *Also let* $\theta_{i}s$ *are relaxations corresponding to the initial contexts* $\phi_{i}s$, *where* 1≤i≤k≤n. *The (multiplicative) tuple closeness* τ(t) *is given by*

$$\tau \left(t\right)=\prod_{i=1}^{k}\chi \left(v_{i},c_{1i},c_{2i}\right)$$

*Finally, for two tuples* $t_{1}$ *and* $t_{2}$, $t_{1}$ *is preferred over* $t_{2}$ *if* $\tau \left(t_{1}\right)\geq \tau \left(t_{2}\right)$ *holds*.

The tuple closeness ranges between 0 and 1. It must be noted here that preference relation is defined only using the relaxation conditions over the attributes involved, i.e., the remaining attributes do not play any role. Given a top-*k* response upper limit, we now can easily choose the top *k* closest responses.

### 4.3 Implementation Considerations

Implementation of ConSQL is ongoing and a full discussion of its implementation is outside the scope of this paper. Issues such as how to map queries to SQL for execution, how to choose preferred tuples as response, how to implement cumulative and disjunctive context switching, are deferred to a later article. In this section we mainly focus on a generic distance function to compute the closeness of a value to help implement the idea of preferred tuples instead.

In general, all relaxations imply acceptable deviations. In CoBase, the relaxation was based on a carefully constructed TAH. Such a structure makes it easier to compute deviations for almost all types of objects or values. For example, we can potentially create a TAH for airports based on GPS locations, overall operation, average delay, or traffic volume, and so many other ways or dimensions. However, as mentioned earlier, developing and maintaining a multi-dimensional TAH is truly complicated, if not impossible.

A practical strategy could be to develop a polymorphic distance function *δ*^*t*^(*x*, *y*, *z*) that is capable of returning the distance between two objects *x* and *y* of type *t* that will range between 0 and 1, relative to *y* within a linear distance between *y* and *z*. This function will measure how close *x* is to *y* given that the object *z* is assumed to be farther than *y* satisfying triangular inequality relationship in a metric space, i.e., *d*(*y*, *z*) ≤ *d*(*y*, *x*) + *d*(*x*, *z*).

## 5 Conclusion

Context is a complex concept to model and serves a unique purpose in various applications. Therefore its definition and application varies. In this article, we have defined context as the intent of a query the response to which we try to approximate as closely as possible. We have leveraged the idea of query relaxation that previously played a major role in designing cooperative query answering systems, and have shown that in chatbot type applications our concept of context can play a significant role in improving conversations and services. The idea presented is at an early stage. The implementation of a query processor is ongoing while its efficacy has been tested as a prototype implementation (see Sec A) by processing the queries by translating them to equivalent SQL queries. Refinement of the model, implementing the polymorphic distance function and context recognition within a text conversation remain as our future research.

## Figures and Tables

**Fig. 3:**
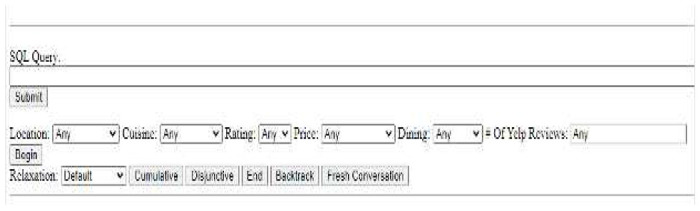
ConSQL front-end.

**Fig. 4:**
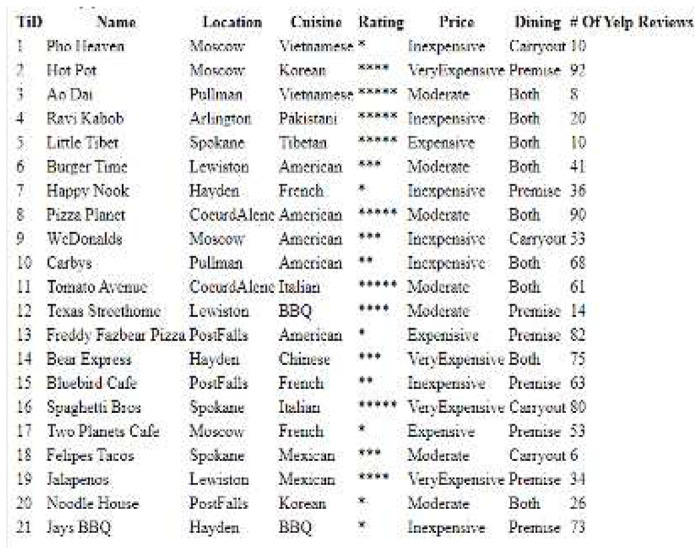
Eaxmple table *restaurants*.

**Fig. 5:**
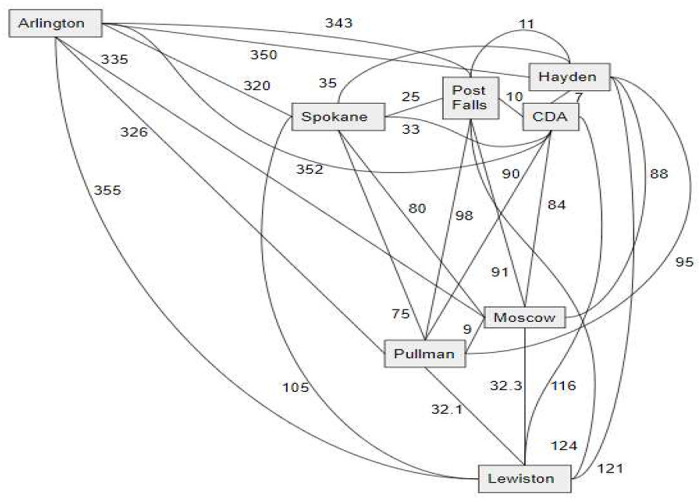
Geo location distance graph in miles for locationproximity distance function.

**Fig. 6:**
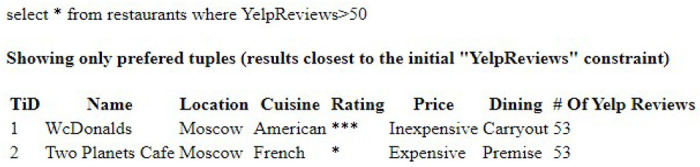
Selected tuples of under “preferred tuples” determination.

**Fig. 7:**
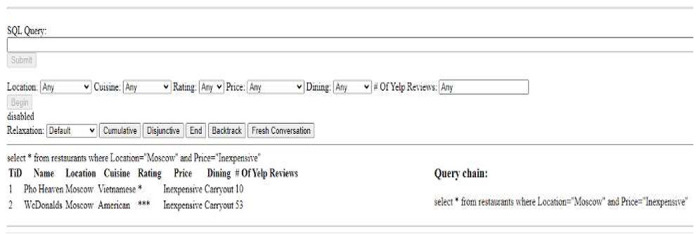
Query execution log shown as query chain.

**Fig. 8:**
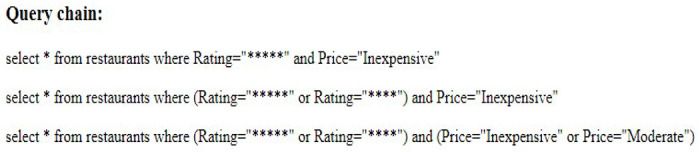
Example cumulative relaxation query chain.

**Fig. 9:**
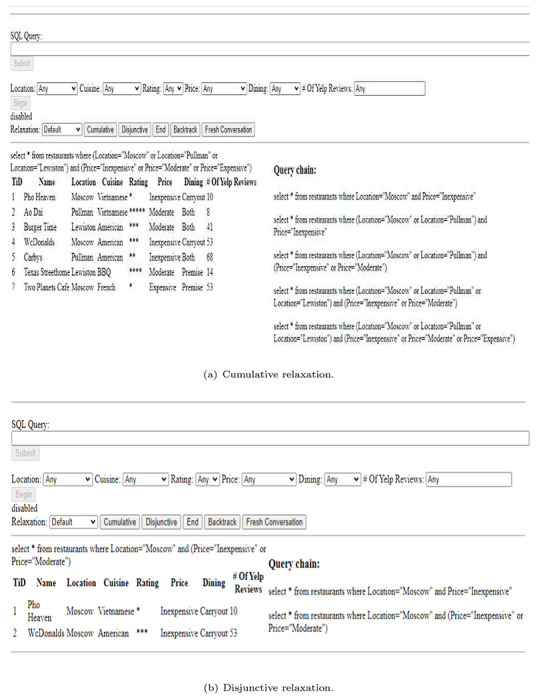
Examples of cumulative and disjunctive relaxations.

## Data Availability

None.

## Footnotes

1
https://chat.openai.com/share/d06aee4d-80f4-4293-8106-9037aa1bf9fa

2 Note that in a session, there can be only one active conversation with multiple initial contexts giving rise to the concept of the context forest.

## A Prototype Implementation

As mentioned in Sec 4.3, a full implementation of ConSQL query processor is ongoing, involved, and outside the scope of this paper. However, we have implemented ConSQL as a graphical user interface driven query processor. In this section, we briefly describe this prototype as a demonstration of ConSQL’s feasibility and efficacy. The discussion to ensue should serve as a proof of concept for ConSQL.

### A.1 Design of ConSQL Prototype Query Processor

The prototype ConSQL query engine has been implemented as a PHP GUI on XAMPP with MySQL as the backend storage and query processing engine. PHP was used for server-side and command line scripting as well as for the desktop GUI. Figures 3 and 4 show respectively the front-end ConSQL engine and an expanded test database as discussed in Sec 1.

The query interface (see Fig 3) lists all the attributes in the example table (or the table being used), and sets a default selection condition “any” as a wildcard. Users are able to choose any condition using the relevant drop down list. It also shows the contextual querying features such as “relaxation”, and relaxation modes such as “cumulative”, or “disjunctive”. Finally it also includes conversation features such “fresh conversation”, “end” and “backtrack” that capture the systax of Con-SQL discussed in Sec 4.1. Users are able to select these as buttons in specific ways. The relaxation drop down menu allows the users to chose which attribute to apply relaxation on. The meaning of the cumulative and disjunctive relaxations follow the semantics discussed in Sec 3.2.

We have implemented distance functions for numeric domains such as price, geographic locations based on location graphs such as the one shown in Fig 5, ratings, and cuisine. The cuisine distance function is based on the height-based ancestral distance as discussed in Sec 1 in reference to Fig 1. These distance functions were used to compute preferred tuples as shown in Fig 6 for a query

 select *

 from restaurants

 where YelpReviews > 3

### A.2 Example Queries

Figure 7 shows a simple query.

 select *

 from restaurants

 where location=‘Moscow’ and Price=‘Inexpensive’

In this figure, a query log called the “query chain” is shown at the right hand side. This log shows the chornology of the queries executed that is reflected in the resultant response table shown to the left. A sample cumulative query chain is shown in Fig 8.

Finally, Fig 9(a) shows the query chain and the result of the query above relaxed four times cumulatively, while Fig 9(b) shows it relaxed twice disjunctively. In these queries, relaxation is encoded in the form of query rewriting. For example, the query above is rewritten as

 select *

 from restaurants

 where (location=‘Moscow’ or location=‘Pullman’ or location=‘Lewiston’) and (price=‘Inexpensive’ or price=‘Moderate’ or price=‘Expensive’)

for the execution shown in Fig 9(a). For the execution in Fig 9(b), it is rewritten as the following query.

 select *

 from restaurants

 where (location=‘Moscow’) and (price=‘Inexpensive’ or price=‘Moderate’)
